# A heritable form of *SMARCE1*-related meningiomas with important implications for follow-up and family screening

**DOI:** 10.1007/s10048-015-0472-y

**Published:** 2016-01-23

**Authors:** E. H. Gerkes, J. M. Fock, W. F. A. den Dunnen, M. J. van Belzen, C. A. van der Lans, E. W. Hoving, I. E. Fakkert, M. J. Smith, D. G. Evans, M. J. W. Olderode-Berends

**Affiliations:** Department of Genetics, University of Groningen, University Medical Center Groningen, P.O. Box 30001, 9700 RB Groningen, The Netherlands; Department of Neurology, Child neurology, University of Groningen, University Medical Center Groningen, Groningen, The Netherlands; Department of Pathology and Medical Biology, Pathology division, University of Groningen, University Medical Center Groningen, Groningen, The Netherlands; Department of Clinical Genetics, Leiden University Medical Center, Leiden, The Netherlands; Department of Neurosurgery, University of Groningen, University Medical Center Groningen, Groningen, The Netherlands; Manchester Centre for Genomic Medicine, Institute of Human Development, Manchester Academic Health Sciences Centre (MAHSC), St. Mary’s Hospital, University of Manchester, Manchester, UK

**Keywords:** *SMARCE1*, Clear cell meningioma, Germline, Tumor predisposition syndrome, Childhood, Hereditary

## Abstract

Childhood meningiomas are rare. Recently, a new hereditary tumor predisposition syndrome has been discovered, resulting in an increased risk for spinal and intracranial clear cell meningiomas (CCMs) in young patients. Heterozygous loss-of-function germline mutations in the *SMARCE1* gene are causative, giving rise to an autosomal dominant inheritance pattern. We report on an extended family with a pediatric CCM patient and an adult CCM patient and several asymptomatic relatives carrying a germline *SMARCE1* mutation, and discuss difficulties in genetic counseling for this heritable condition. Because of the few reported cases so far, the lifetime risk of developing meningiomas for *SMARCE1* mutation carriers is unclear and the complete tumor spectrum is unknown. There is no surveillance guideline for asymptomatic carriers nor a long-term follow-up recommendation for *SMARCE1-*related CCM patients as yet. Until more information is available about the penetrance and tumor spectrum of the condition, we propose the following screening advice for asymptomatic *SMARCE1* mutation carriers: neurological examination and MRI of the brain and spine, yearly from diagnosis until the age of 18 and once every 3 years thereafter, or in between if there are clinical symptoms. This advice can also be used for long-term patient follow-up. More data is needed to optimize this proposed screening advice.

## Introduction

Meningiomas mostly arise in middle-aged people. Occurrence in childhood is rare [1]. Meningiomas account for a small subset (1–4 %) of all pediatric brain tumors [2]. A meningioma in childhood can be the first presenting symptom of neurofibromatosis type 2 (OMIM 101000), caused by mutations in the *NF2* gene [3–5]. Our knowledge of the etiology of meningiomas not caused by neurofibromatosis type 2 is still limited [6]. Some meningiomas are caused by germline mutations in the *SMARCB1* gene, but here the risk for single meningiomas without the occurrence of schwannomas is rare [7, 8]. Meningiomas may also occur due to germline mutations in *SUFU* [9, 10].

One subtype of meningiomas, the clear cell subtype, arises more frequently in young people compared to more common subtypes of meningiomas [11]. Clear cell meningiomas (CCM) are a subtype with a specific histology and in situ behavior. The tumors are more aggressive with a tendency to recur and metastasize within the CNS compared to nonclear cell meningiomas. In the World Health Organization classification of “tumors of the CNS,” clear cell meningiomas are defined as grade 2 because of their aggressiveness [12]. Early detection and treatment are therefore of paramount importance for this tumor type.

Recently, marked steps in the etiologic understanding of clear cell meningiomas were taken. In 2013 and 2014, Smith et al. reported on heterozygous germline mutations in the *SMARCE1* gene in 16 patients from 11 unrelated families with spinal and intracranial CCM [11, 13, 14]. The patients were mostly children, adolescents, or young adults. The first mutations were detected after whole-exome sequencing and further cases were proven by Sanger sequencing. In the examined tumors, loss of the SMARCE1 protein was shown by immunohistochemical analysis. Tumor DNA showed loss of heterozygosity (LOH) of the wild-type allele or a second inactivating mutation as a second hit in some tumors, implying a tumor suppressor function of the *SMARCE1* gene. These findings prove the existence of a hereditary tumor predisposition syndrome with an increased risk for spinal and intracranial CCMs (OMIM 607174). Genetic testing and counseling in afflicted families have now become possible by finding the causative gene but poses new questions and difficulties because of the sparse knowledge so far.

Here we present a family with a pediatric CCM patient and an adult CCM patient and several asymptomatic relatives carrying a germline *SMARCE1* mutation. We propose a screening advice for asymptomatic carriers in the family and for long-term patient follow-up.

## Case report

**Fig. 1:**
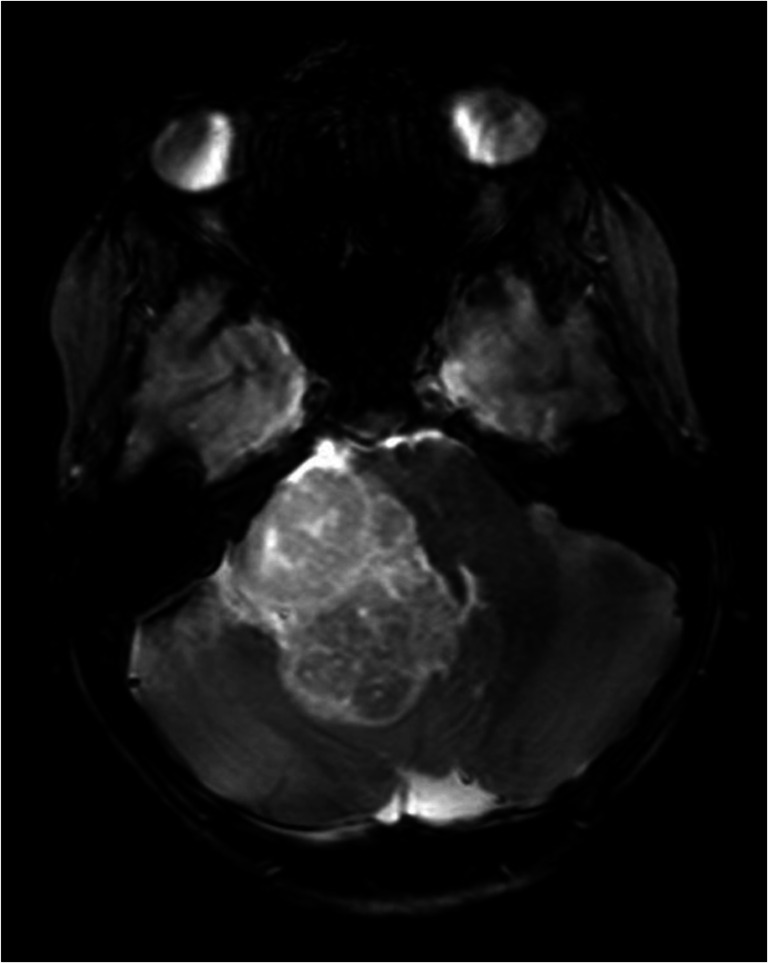
Heterogenous tumor within the right cerebello-pontine angle causing severe compression of the brainstem. This T2-weighted MRI image in the transversal plane illustrates the extrinsic nature of the lesion with a differential diagnosis of meningioma or Schwannoma

**Fig. 2:**
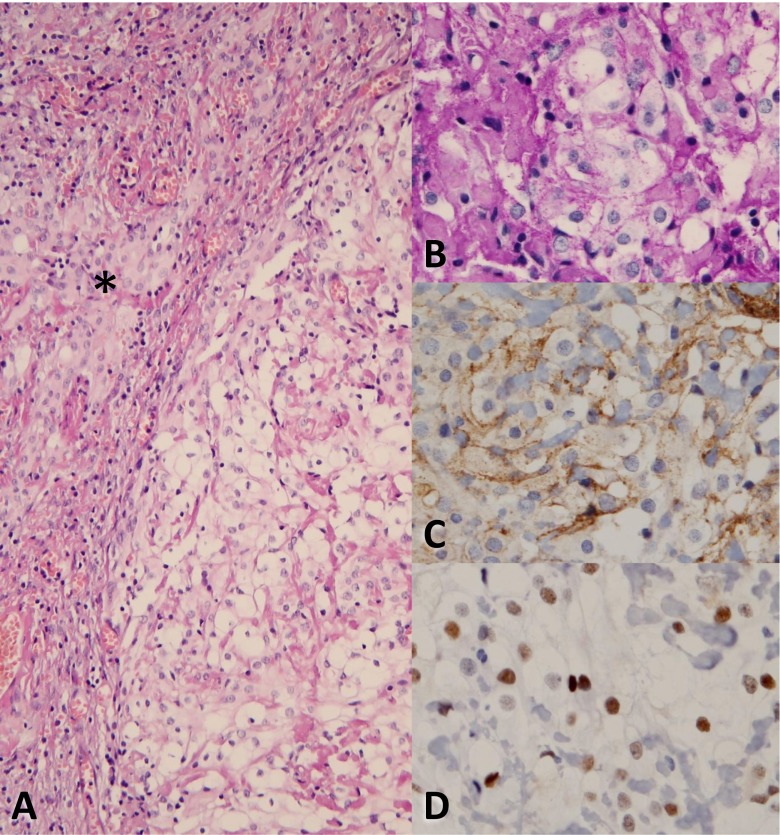
H&E staining of the tumor (**a**, magnification ×20), partly consisting of clear cells on the right side. The *asterisk* indicates meningothelial cells. In (**b**) to (**d**) more detailed micrographs (magnification ×40) of the clear cell component after PAS, EMA and progesterone receptor staining, respectively

A 10-year-old boy was referred to our centre because of recent onset of hearing loss and tinnitus of the right ear. He complained about blurry vision. His medical history was unremarkable apart from treatment with methylphenidate because of ADHD. Physical examination of the ear, nose and throat showed no abnormalities apart from an abnormal Weber test to the left, and an asymmetric reaction of facial nerve. The audiogram showed a sensorineural hearing loss of the right ear, with a downsloping audiogram and complete loss of higher tones indicating damage to the acoustic nerve. The MRI scan of the brain showed a large extrinsic tumor in the right cerebello-pontine angle with severe compression and displacement of the brainstem (Fig. 1). The tumor could be removed in two successive surgical sessions. In the first surgery, the tumor mass could be taken out almost completely except for a very adherent remnant on the vertebral artery and a second separate tumor on the other side. In spite of the close involvement of the lower cranial nerves, all these nerves could be saved anatomically and functionally as monitored intraoperatively. Pathological examination of the tumor showed a clear cell type meningioma, WHO grade II (Fig 2). Hereafter, a second surgery with the aim of radical resection of the remnants was undertaken with good results (Simpson classification I). Post-operatively, the patient experienced swallowing difficulties due to multiple cranial nerve apraxia. In due time, he recovered well and, after 4 months, he was able to speak, eat and drink normally while some atrophy of the right part of the tongue remained. There has been no local recurrence of tumor during 1-year follow-up.

**Fig. 3:**
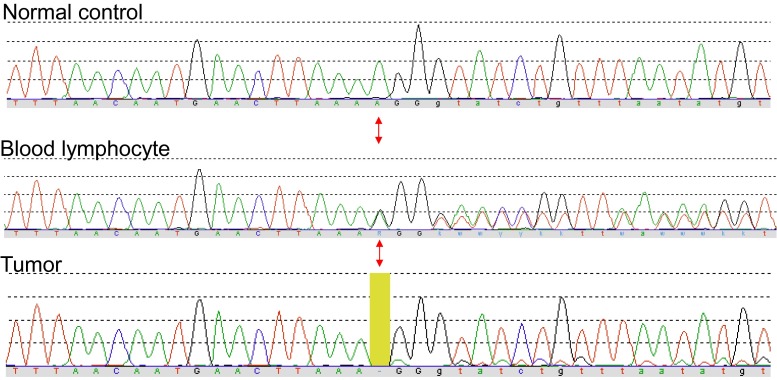
DNA sequencing chromatograms from the patient’s blood lymphocyte DNA and tumor DNA from fresh frozen tissue, and a normal control for reference. The mutation locus of the SMARCE1 c.814delA frameshift mutation is indicated by the *red arrows*. In blood lymphocyte (germline) DNA, the mutation is present in heterozygous state, while in the tumor, it is present in homozygous state, indicating loss-of-heterozygosity (LOH) at the mutation locus. The minimal amount of wild-type sequence that is visible in the tumor sample is caused by a small amount of normal tissue mixed with the tumor cells (color figure online)

Sanger sequencing of the *SMARCE1* gene in DNA from blood showed a 1-bp deletion causing a frameshift in exon 9: c.814delA, p.Arg272Glyfs*5. This particular mutation was not reported before, but the nature of the mutation makes it very likely pathogenic. Analysis of tumor DNA showed homozygosity of this mutation indicating loss-of-heterozygosity (LOH) at the mutation locus (Fig 3). Mutation analysis of the *NF2* gene was normal.

### Family history

**Fig. 4:**
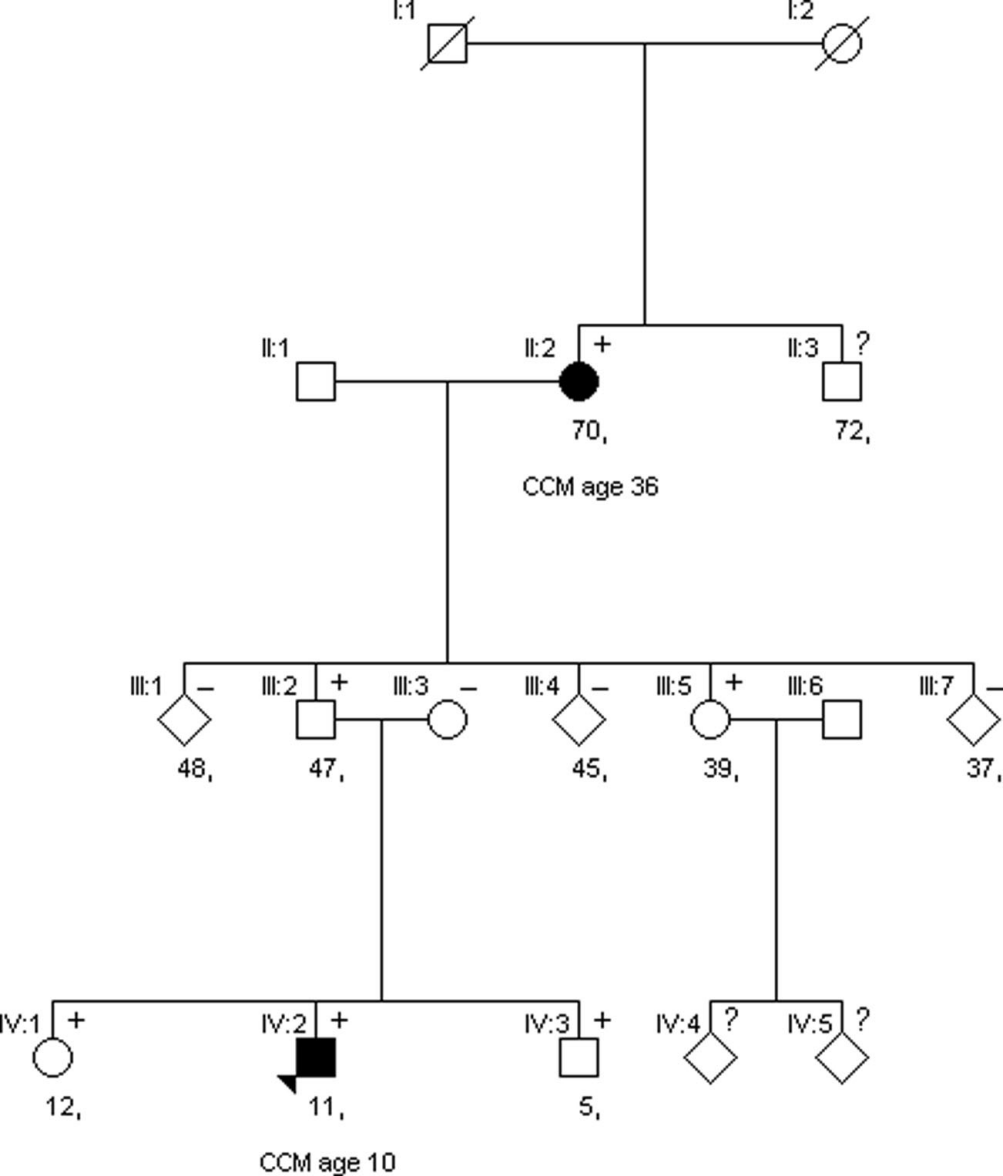
Pedigree of the family with the *SMARCE1* mutation. Current age is mentioned below the *square*/*circle*. + = mutation positive, − = mutation negative. *Solid black* = CCM patient, age of detection of CCM is mentioned below the current age. *Solid white* = clinically asymptomatic.? = testing not started yet

The parents of the patient were tested for the *SMARCE1* mutation after genetic counseling. The father was shown to be a carrier. An MRI brain and spine showed no tumors. As shown in the pedigree, the paternal grandmother had a spinal tumor removed at age 36. The initial pathology report mentioned an ependymoma, but revision showed a clear cell meningioma grade II. Recent follow-up with MRI showed no abnormalities at age 70. The brother and sister of the index case and a paternal aunt proved to be asymptomatic carriers after presymptomatic testing. All were referred for neuroaxis screening and MRI of the brain and spine in follow-up. A recent MRI brain and spine showed no abnormalities in the aunt. MRI brain of the brother at age five because of temporary diplopia showed no abnormalities. The further MRI results of the brother and sister are pending. The family pedigree is shown in Fig. 4.

## Discussion

This case report shows further evidence for the role of *SMARCE1* mutations in the etiology of clear cell meningiomas. We address here the following questions: why to screen, who to screen, what to screen and how to screen in afflicted families and discuss the difficulties in determining what is the best practice.

### Why to screen?

Early detection of meningiomas is of highest importance to prevent damage of surrounding tissue and dissemination. It also enhances the chance of total radical resection. For childhood and adolescent meningiomas, the extent of the initial surgical resection is the strongest independent prognostic factor [15]. Radical neurosurgical resection of very large tumors in eloquent areas may be more hazardous to the patient with a greater chance of (transient) post-operative morbidity, as illustrated in our case. Clear cell meningiomas tend to behave more aggressively and have a tendency to recur and metastasize, making it even more paramount to discover this type as early as possible [12]. Unfortunately, meningiomas frequently cause symptoms late, only after they have grown slowly to a large size. The mass effect of the lesion on the environment finally creates symptoms, like in our patient. This may interfere with early detection. Screening in high-risk groups could therefore be beneficial.

### Who to screen?

Patients that have been treated for a meningioma will remain in follow-up for several years but are discharged thereafter because the risk of recurrence will decline. However, patients with a history of a *SMARCE1-*related meningioma will probably remain at a higher risk for further CCMs, particularly if female. This makes them a possible high-risk group for which a long-term follow-up schedule might be beneficial. It is currently unknown whether the risk for CCMs remains increased during the rest of the lifetime or decreases substantially after a certain age, as in NF2 where the risk for new tumors declines with older age [16]. We believe that long-term screening is advisable until we have more knowledge on this.

Carrier family members could constitute a second high-risk group for CCMs. Presymptomatic testing of family members after genetic counseling can be beneficial because it allows for justified screening and early tumor detection in carriers. However, genetic counseling is complicated by the fact that the penetrance of the disease and the lifetime risk for CCMs are unknown at the moment. Moreover, no established guideline is available providing a screening advice. Penetrance of the disease could be overestimated based on current literature, as only a few families have been described so far with probably a detection bias towards families with more than one patient. Several asymptomatic carriers of different ages and gender were detected in the family reported here. Incomplete penetrance for meningiomas in *SMARCE1* mutation- positive families was shown before by Smith et al. They found three asymptomatic male carriers, aged 17, 71, and middle age, coming from three different families with more than one meningioma patient [13, 14].

### What to screen?

There is no certainty about the complete tumor spectrum of this tumor syndrome as of yet. We know from other autosomal dominant hereditary cancer predisposition syndromes that tumors can arise in different and distinct tissue types, for example, in Lynch syndrome and Von Hippel Lindau syndrome [17, 18]. In those diseases, the problem is bi-allelic shutdown of a tumor suppressor gene within the tumor tissue. It apparently depends on the nature of the exact tumor suppressor gene, which types of tissues will be at risk for tumor formation. The evidence of the few cases of *SMARCE1*-related CCMs that have been published so far, together with the evidence of the patient reported here, shows that the *SMARCE1* gene also acts as a tumor suppressor gene [13]. In the clear cell meningiomas, there is a second hit causing inactivation of the wild-type allele [14]. So far, no other tumor types than spinal and intracranial CCMs have been described in *SMARCE1*-positive patients. Strategic gathering of patient and family data can help determine if we need to be on the lookout for other types of tumors in carriers, and further knowledge is needed to better understand why loss of SMARCE1 expression specifically leads to CCMs. For now, we propose to screen for spinal and intracranial CCMs only. Raffalli-Ebezant et al. reported on a carrier female who appeared to have multiple, asymptomatic spinal lesions in keeping with intradural meningiomas [11]. This suggests that multiple tumors can be present and screening of the whole brain and spine is advisable.

All mutations in the *SMARCE1* gene found so far in CCM patients are loss-of-function mutations, including frameshift and nonsense mutations, an inversion and two large deletions [11, 13, 14, 19]. Missense mutations in the *SMARCE1* gene cause a clinically very different syndrome called Coffin-Siris syndrome (OMIM 135900) with congenital mental retardation and dysmorphisms as main features [20, 21]. The so far known CCM patients with a loss-of-function *SMARCE1* mutation have no clinical signs of Coffin-Siris syndrome, and screening for developmental delay or dysmorphisms is therefore not necessary.

It is currently unclear if missense mutations in the *SMARCE1* gene causing Coffin-Siris syndrome predispose to CCMs later in life. This combination has actually been described very recently for a related gene causing Coffin-Siris syndrome, the *SMARCB1* gene. A patient with Coffin-Siris syndrome phenotype and a constitutional missense *SMARCB1* gene mutation developed schwannomatosis [22].

### How to screen?

Until more information is available about the penetrance and complete tumor spectrum of the condition, we propose the following screening advice for asymptomatic *SMARCE1* mutation carriers: neurological examination and MRI of the brain and spine, yearly from diagnosis until the age of 18 and once every 3 years thereafter, or in between if there are clinical symptoms.

For asymptomatic carrier children/adolescents, more frequent neurological screening is advised than for adults, because they are less well equipped to detect and mention any neurological problems and because the risk to develop tumors in *SMARCE1* carriers seems to be larger at younger age, especially for boys [11, 13, 14, 19]. Furthermore, childhood meningiomas in general tend to have a more aggressive biological behavior and a worse prognosis than the same tumors in adults, justifying a more aggressive screening approach in children to detect the tumors in an early state [1, 23, 24]. A disadvantage of starting screening at a young age is the fact that MRI of the neuraxis in young or noncooperative patients requires sedation or general anesthesia because of the long duration of the imaging. The use of melatonin as a low-risk sedation substitute could help prevent this in a subset of patients. The morbidity and mortality risk of sedation or anesthesia in children is small if done in a well-equipped centre with experience.

All symptomatic males with a *SMARCE1* mutation described so far developed meningiomas in childhood (age range 2–10 years), while the symptomatic carrier females developed tumors somewhat later in adolescence or early adulthood (age range 14–30s) [11, 13, 14, 19]. This was again shown in our family. These findings suggest that penetrance of the disease might be age- and gender-dependent. Those carrier males who do develop a tumor seem to develop it at early age and females seem to be at higher risk after onset of their fertile period. The increased penetrance for males for meningiomas in childhood is also found both in sporadic meningiomas and NF2 [5] with the reverse being true in adulthood. Smith et al. hypothesize a possible hormonal influence on penetrance, but there is no scientific proof on this as yet [10, 13]. We believe that this ample amount of evidence for gender- and age-specific penetrance is not enough to adjust a screening advice depending on age or gender at the present time. If the evidence becomes stronger, less surveillance for men after a certain age could possibly be considered.

For patients after meningioma treatment, frequency and duration of the follow-up surveillance depend on the location of the tumor, the WHO grade of the tumor, and the extent of the tumor resection according to the Dutch national guideline Intracranial Meningioma [25]. The above mentioned screening advice could be used for long-term follow-up thereafter for *SMARCE1-*related CCM patients, because of their enlarged risk of additional primary tumors.

### Future directives

Hopefully, substantiated adjustment to this proposed screening advice can be made after structural gathering of more medical and genetic data of patients and families. This will help to better estimate the age- and gender-dependant penetrance of the disease and gain more certainty about the complete tumor spectrum for carriers of a *SMARCE1* mutation. The addition of this report about a larger family to the current literature on the subject hopefully is a step in the right direction, raising awareness of the condition and adding relevant data to the knowledge.
